# Genome-Wide Association Study of Gene by Smoking Interactions in Coronary Artery Calcification

**DOI:** 10.1371/journal.pone.0074642

**Published:** 2013-10-03

**Authors:** Linda M. Polfus, Jennifer A. Smith, Lawrence C. Shimmin, Lawrence F. Bielak, Alanna C. Morrison, Sharon L. R. Kardia, Patricia A. Peyser, James E. Hixson

**Affiliations:** 1 Division of Epidemiology, Human Genetics and Environmental Sciences, The University of Texas Health Science Center at Houston, Houston, Texas, United States of America; 2 Department of Epidemiology, University of Michigan, Ann Arbor, Michigan, United States of America; Johns Hopkins University, United States of America

## Abstract

Many GWAS have identified novel loci associated with common diseases, but have focused only on main effects of individual genetic variants rather than interactions with environmental factors (GxE). Identification of GxE interactions is particularly important for coronary heart disease (CHD), a major preventable source of morbidity and mortality with strong non-genetic risk factors. Atherosclerosis is the major cause of CHD, and coronary artery calcification (CAC) is directly correlated with quantity of coronary atherosclerotic plaque. In the current study, we tested for genetic variants influencing extent of CAC via interaction with smoking (GxS), by conducting a GxS discovery GWAS in Genetic Epidemiology Network of Arteriopathy (GENOA) sibships (N = 915 European Americans) followed by replication in Framingham Heart Study (FHS) sibships (N = 1025 European Americans). Generalized estimating equations accounted for the correlation within sibships in strata-specific groups of smokers and nonsmokers, as well as GxS interaction. Primary analysis found SNPs that showed suggestive associations (p≤10^−5^) in GENOA GWAS, but these index SNPs did not replicate in FHS. However, secondary analysis was able to replicate candidate gene regions in FHS using other SNPs (+/−250 kb of GENOA index SNP). In smoker and nonsmoker groups, replicated genes included *TCF7L2* (p = 6.0×10^−5^) and *WWOX* (p = 4.5×10^−6^); and *TNFRSF8* (p = 7.8×10^−5^), respectively. For GxS interactions, replicated genes included *TBC1D4* (p = 6.9×10^−5^) and *ADAMTS9* (P = 7.1×10^−5^). Interestingly, these genes are involved in inflammatory pathways mediated by the NF-κB axis. Since smoking is known to induce chronic and systemic inflammation, association of these genes likely reflects roles in CAC development via inflammatory pathways. Furthermore, the NF-κB axis regulates bone remodeling, a key physiological process in CAC development. In conclusion, GxS GWAS has yielded evidence for novel loci that are associated with CAC via interaction with smoking, providing promising new targets for future population-based and functional studies of CAC development.

## Introduction

Recent genome-wide association studies (GWAS) have identified numerous novel loci associated with common diseases and their risk factors. However, GWAS have typically focused only on main effects of individual genetic variants, rather than interactions with other genes (epistasis) and with environmental factors (GxE interactions). Although GxE interactions provide a well-established paradigm for progression of complex chronic diseases, more precise biological and statistical characterization of this interplay remains elusive [1]. Identification of GxE interactions is particularly important for coronary heart disease (CHD), a major preventable source of morbidity and mortality with strong non-genetic risk factors such as physical activity, diet, and smoking.

Atherosclerosis is the major cause of CHD, and extent of coronary atherosclerosis is the most powerful predictor of subsequent clinical events. Non-invasive imaging of coronary artery calcification (CAC) has emerged as a useful method to assess CHD risk. The quantity of CAC, measured by computed tomography (CT), is heritable [2], [3]. and correlates directly with the quantity of coronary atherosclerotic plaque. Furthermore, CAC scores predict all cause mortality [4] and coronary outcomes in asymptomatic individuals as shown in a cohort of over 10,000 individuals followed for 5 years [5], [6]. A CAC score >100 has demonstrated clinical relevance representing the transition from mild to moderate coronary atherosclerosis [7], [8]. Additionally, a CAC score >100 is associated with a 7 fold increased risk for myocardial infarction (MI) and CHD death after adjusting for traditional risk factors [9].

A recent CAC GWAS conducted by the Cohorts for Heart and Aging Research in Genomic Epidemiology consortium (CHARGE) included five independent cohorts for discovery and three cohorts for replication [10]. The strongest SNP associations for CAC quantity and score >100 were found on chromosome 9p21 (top SNPs near *CDKN2A* and *CDKN2B*) and within the *PHACTR1* gene on chromosome 6p24. These same regions are associated with early CHD. To date, no GWAS has investigated associations between GxE interactions and CAC.

Cigarette smoking, a major risk factor for CHD, is associated with CAC. In a study of over 30,000 asymptomatic adults, Hoff et al. reported a significant, independent association between having ever smoked and CAC score >100 (OR = 1.8 in men and 1.5 in women) [11]. Recently, North et al. found several chromosomal regions with evidence for linkage with CAC quantities only in nonsmokers (chromosomes 4 and 6) or only in smokers (chromosomes 11 and 13), with significant genotype by smoking interactions (p<0.05) [12].

In the current study, we used GWAS to test for genes that interact with smoking for CAC score >100 in European Americans from the Genetic Epidemiology Network of Arteriopathy study (GENOA). Our primary analysis included GWAS analysis of GENOA subgroups stratified by smoking status, as well as genome-wide tests for variants that show significant Gene by Smoking interactions (GxS). Primary analysis also attempted to replicate GENOA index SNPs that showed suggestive associations (p<10^−5^) in FHS. For secondary analysis, we tested SNPs located in candidate gene regions (+/−250 kb of GENOA index SNP) for associations with CAC in the Framingham Heart Study (gene-based strategy).

## Methods

### Ethics Statement

These studies received approval from Institutional Review Boards (University of Texas Health Science Center at Houston IRB, University of Michigan Health Sciences and Behavioral Sciences IRB, Boston University IRB), and study participants gave written informed consents.

### Characteristics of Study Cohorts

The discovery cohort consisted of sibships of European ancestry who participated in the GENOA study as part of the NHLBI Family Blood Pressure Program (FBPP) (FBPP Investigators, 2002). Sibships containing at least two individuals diagnosed with essential hypertension before age 60 years were recruited from Rochester, Minnesota. All other siblings were invited to participate regardless of hypertensive status. Exclusion criteria were secondary hypertension, alcoholism or drug abuse, pregnancy, insulin-dependent diabetes mellitus, or active malignancy. GENOA sibships recruited during Phase 1 (N = 1,583, during 1995 to 2000) were invited to participate in Phase 2 (N = 1,241, during 2000 to 2004) and received electron beam CT scans of the heart. Other information collected by the GENOA study included demographic, environmental, anthropometric, and physiological data. Participant measurements of blood pressure and other clinical and physiological data have been described elsewhere [10]. Individuals with a history of coronary revascularization (n = 83) were excluded from measurement of CAC. Participants with a history of myocardial infarction (n = 19), stroke (n = 27), positive angiogram (n = 31), missing data (n = 2), or self-reporting as Hispanic ancestry (n = 2) were excluded from analyses. Of the 1,077 GENOA participants with CAC and risk factor measures, 915 participants (539 women and 376 men in 421 sibships) had genotype data.

The replication study participants were European Americans from the Offspring Cohort of the Framingham Heart Study (FHS) that participated in the Offspring Exam 7 (n = 1,314) conducted between 1998 and 2001. FHS participants were excluded from the analyses if considered to have a cardiovascular disease (CVD) event prior to Exam 7 (N = 160) yielding the final number of siblings with valid genotype data to 1,025 participants in 431 sibships. A CVD event was defined as the occurrence of coronary death, myocardial infarction, stable or unstable angina pectoris, atherothrombotic stroke, intermittent claudication, or cardiovascular death, and hospitalized coronary insufficiency. Risk factors were assessed from participants undergoing a routine physical examination, anthropometry, and laboratory collection during offspring examination [10].

### GWAS Genotyping

GENOA participants were genotyped with the Affymetrix Genome-Wide Human SNP Array 6.0. A total of 669,293 SNPs were genotyped in the 915 participants after passing quality control measures including exclusion of SNPs with a call rate <95%, or a minor allele frequency (MAF) <0.01. These measured SNP genotypes were used for imputation (CEU HapMap haplotypes, release 22, build 36) by Markov Chain modeling (MACH version 1.0), yielding 2,171,047 SNPs for subsequent GWAS analyses (excluded SNPs with MAF <0.05) [13], [14].

A total of 8,481 FHS participants were genotyped using the Affymetrix GeneChip Human Mapping 500K Array and an additional 50K Affymetrix gene-focused Molecular Inversion Probe (MIP) array. After excluding SNPs with a call rate <97% or MAF <0.01, 378,163 SNPs were used for imputation (CEU HapMap haplotypes, release 22, build 36) with MACH version 1.0.15, yielding 2,543,887 SNPs.

### CAC Measurements

GENOA participants were imaged with an Imatron C-150 electron beam CT scanner (Imatron Inc.) as previously described (O'Donnell et al., 2011). Scan results were initially reviewed by a radiologist for technical quality, and then scored by a radiologic technologist. A focus of CAC was considered to exist if there were at least four contiguous pixels ≥130 Hounsfield Units (HU) in density. The CAC score is a calculation based on the number, area, and density of CAC foci summed from the four major epicardial arteries using the method of Agatston et al. (1990).

For FHS participants, a multidetector CT exam was conducted between 2002 to 2005 with a calcified lesion in the coronary arteries defined as an area of at least 3 contiguous pixels >130 HUs with the use of 3-dimensional connectivity criteria (6 points). Agatson scores developed for electron beam CT scans were modified for multidetector CT scans as described previously (Parikh 2007). A CAC score cutpoint of 100 to define a qualitative outcome was used in all analyses and is referred to as CAC.

### Categorization of smoking status

Smokers (current and previous) and nonsmokers (never) were classified based on self-report. Previous smokers were categorized by having smoked more than 100 cigarettes in a lifetime according to the National Health Interview Survey [15]. Self-reports were used for classification since biochemical measures (e.g., cotinine levels) of smoking amounts were not available for GENOA participants. A dichotomous categorization of smoking status rather than a quantitative measure (e.g., pack-years) was chosen due to the inherent high dimensionality of GWAS analysis (2.1 million SNPs with 4.2 million main effects and interaction variables per SNP).

### Data Analysis

In order to account for the correlation among sibships, the Genome-Wide Association Analysis with Family (GWAF) package was utilized within R statistical software version 2.10 employing a generalized estimating equations (GEE) model [16]. Adjustment for additional covariates in the GEE model included age, sex, body mass index (BMI), pulse pressure, diabetes, systolic blood pressure (SBP), use of anti-hypertensive medications, use of lipid lowering medications, and LDL-cholesterol. To assess population stratification in GENOA GWAS, the first ten principal components (PC) were calculated using Eigenstrat [17]. However, none of the PCs were significant (p<0.05) in GEE models (p = 0.82 for the first PC), so were not used in subsequent analyses. To assess interactions of genotype by smoking (GxS) status, subgroups of smokers/nonsmokers were analyzed separately. Results from the stratified analyses were then used to test for interaction in the combined group. Effect differences for smoking were assessed by testing if beta coefficients differed from zero for associations of individual SNPs with CAC. The test for interaction approximates a 2 sample t-test with the following null hypotheses:

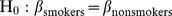

where β_smokers_ is the beta coefficient for the SNP in smokers and β_nonsmokers_ is the beta coefficient for the SNP in nonsmokers.

For all 2.1 million discovery SNPs, the interaction test statistic was calculated from the following equation as adapted from Heid et al. [18].

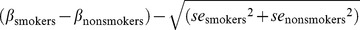




Primary analysis started with GxS GWAS in GENOA sibships, followed by testing of index SNPs that showed suggestive evidence for association (p≤10^−5^) in FHS sibships. A genome-wide significance threshold based on Bonferroni correction was used to account for multiple testing of 2.1 million SNPs (p≤2.3×10^−8^). Analyses in FHS were carried out within the corresponding strata-specific/interaction subgroups as described in GENOA. Secondary analysis used a gene-based approach that tested multiple SNPs in FHS sibships that were located in associated gene regions (+/−250 kb of GENOA index SNPs with GWAS p≤10^−5^). We also considered genes containing SNPs with p-values between 10^−4^ and 10^−5^, and that were near genes (≤250 kb) with functions related to CAC (NCBI MeSH search). For secondary analysis in FHS, we accounted for multiple testing of correlated SNPs within each gene region using LD-based Bonferroni corrections with the number of LD blocks centered around the GENOA discovery SNP (significance threshold of p≤0.05/number of blocks in each gene region) [19]. LD blocks were delimited by SNPs in strong LD (95% with D′≥0.70) according to Haploview (CEU HapMap Build 22) [20].

## Results

GENOA study participants were in 421 sibships ranging in size from 1 to 10, with the majority of sibships consisting of size 2 (44%) or size 3 (21%). The general characteristics of the GENOA participants stratified by gender and smoking status are presented in Table 1. Overall, more participants were female (59%) than male (41%), and males had a higher proportion of smokers (58%) compared to females (38%). Participants in all categories had similar ages (mean age of 58.1 years). Males had higher mean CAC scores than females, regardless of smoking status. Smokers had higher mean CAC scores than nonsmokers in both men (341 versus 271) and women (150 versus 84). Table 1 also shows general characteristics for the stratified FHS participants. Overall, FHS participants were older than GENOA participants, with lower use of hypertensive and lipid-lowering medications, and lower frequency of diabetes. Like GENOA, smokers had higher mean CAC scores in both men (468 versus 238) and women (107 versus 81).

**Table 1:** Stratified descriptive statistics by gender and smoking for the discovery cohort (Genetic Epidemiology Network of Arteriopathy Study, GENOA) and the replication cohort (Framingham Heart Study).

	GENOA					
	Males (N = 376)			Females (N = 539)		
	Smoker (N = 219)	Non-Smoker (N = 157)	P value	Smoker (N = 207)	Non-Smoker (N = 332)	P value
CAC>100 n(%)^a^	103 (69.1)	64 (54.7)	0.228	46 (30.9)	53 (45.3)	0.068
CAC≤100 n(%)	116 (41.9)	93 (25.0)		161 (58.1)	279 (75.0)	
CAC score	340.8	270.5		150.1	83.5	
Log (CAC +1)^b^	3.82±2.6	3.55±2.5	0.30	2.67±2.5	1.96±2.2	0.05
Age (years)	58.7±9.7	58.0±10.9	0.530	56.6±9.4	58.7±10.5	0.197
LDL-C (mg/dL)	113.3±27.7	122.8±31.8	0.002	116.3±30.5	115.9±32.0	0.868
BMI (kg/m^2^)	30.8±5.1	30.7±5.1	0.829	30.5±7.6	30.8±6.6	0.685
Pulse Pressure	54.2±14.1	53.2±14.9	0.536	56.0±14.4	59.7±16.4	0.008
Diabetes n(%)	41 (18.7)	19 (12.1)	0.084	24 (11.6)	39 (11.8)	0.957
Hypertensive Meds n(%)	140 (63.9)	102 (65.0)	0.835	136 (65.7)	225 (67.8)	0.619
Lipid Lowering Meds n(%)	77 (35.2)	43 (27.4)	0.111	43 (20.8)	67 (20.2)	0.868

	FRAMINGHAM HEART STUDY					
	Males (N = 459)			Females (N = 566)		
	Smoker (N = 259)	Non-Smoker (N = 200)	P value	Smoker (N = 309)	Non-Smoker (N = 257)	P value
CAC>100 n(%)	136 (52.5)	80 (0.40)	0.008	66 (21.4)	50 (19.5)	0.576
CAC≤100 n(%)	123 (47.5)	120 (0.60)		243 (78.6)	207 (80.5)	
CAC score	467.9	238.2		106.5	81.3	
Log (CAC +1)	4.31±2.51	3.22±2.55	<0.001	2.30±2.37	1.99±2.27	0.106
Age (years)	63.8±8.8	60.9±9.4	0.001	62.6±8.1	64.1±9.3	0.038
LDL-C (mg/dL)	119.5±31.3	122.9±29.0	0.229	121.9±36.1	119.6±30.7	0.408
BMI (kg/m^2^)	28.9±4.3	28.1±4.2	0.057	27.8±5.7	27.2±5.3	0.195
Pulse Pressure	50±13.1	48.5±14.3	0.258	49.6±15.2	51.0±15.0	0.257
Diabetes n(%)	21 (8.1)	11 (5.5)	0.277	17 (5.5)	18 (7.0)	0.460
Hypertensive Meds n(%)	80 (30.9)	58 (29.0)	0.662	86 (27.8)	74 (28.8)	0.802
Lipid Lowering Meds n(%)	39 (15.1)	30 (15.0)	0.986	37 (12.0)	39 (15.2)	0.266

CAC, coronary artery calcification; LDL-C, Low Density Lipoprotein-Cholesterol; BMI, body mass index.

a For categorical variables, the number and percent are presented (Chi-square P-values).

b For continuous variables, the mean and standard deviation are presented (t-test P-values).

Table 2 presents a list of index SNPs and their nearest genes that reached p≤10^−5^ separately for smokers and nonsmokers in GENOA, and for GxS interactions. Primary analysis also included attempts to replicate our discovery results (index SNPs with p≤10^−5^) in the FHS cohort. Table 2 shows corresponding p values for GENOA index SNPs in FHS sibships. Overall, only one SNP (*COLEC11* rs12990669 in GENOA smokers, p = 1.4×10^−8^) reached genome-wide significance levels (p≤2.3×10^−8^) after Bonferroni corrections for multiple testing in GENOA or FHS (Table 2). However, this signal may represent a false positive result as other SNPs in *COLEC11* did not show associations.

**Table 2:** Primary analysis: SNPs that showed genome-wide associations (p≤1.0×10^−5^) with CAC in GENOA (smokers, nonsmokers, gene×smoking interactions).

*Smokers*									
Locus			Association						Nearby Genes
SNP	Chr	Position (Mb)	Allele (+/−)	MAF	Beta	SE	GENOA P value	FHS P Value	Relative position (−upstream,+downstream)
rs12990669	2	3649241	(C/G)	0.07	−1.79	0.32	1.36×10^−8^	0.71	*COLEC11*
rs2190305	7	28859450	(G/A)	0.19	−0.98	0.20	1.24×10^−6^	0.06	*CREB5*
rs10131267	14	30868736	(G/A)	0.08	−1.40	0.29	1.77×10^−6^	0.44	*intergenic*
rs9574536	13	80585091	(C/T)	0.34	1.41	0.30	2.44×10^−6^	0.67	*intergenic*
rs8047995	16	78949736	(G/C)	0.32	1.13	0.25	4.51×10^−6^	0.66	*WWOX*
rs7158225	14	84397780	(T/C)	0.21	−0.89	0.20	5.34×10^−6^	0.69	*intergenic*
rs17608293	18	13378636	(A/G)	0.38	0.83	0.18	6.01×10^−6^	0.77	*C18ORF1*
rs7926081	11	4676977	(G/T)	0.49	−0.83	0.19	8.51×10^−6^	0.92	*intergenic*
rs7988945	13	51496320	(A/G)	0.39	0.96	0.22	8.94×10^−6^	0.44	*RNASEH2B*
rs7411138	1	240716461	(C/A)	0.10	1.34	0.33	5.66×10^−5^	0.10	*GREM2*
rs7089541	10	68558800	(A/G)	0.19	−0.96	0.24	5.77×10^−5^	0.54	*CTNNA3*
rs10128255	10	114742835	(G/A)	0.36	0.82	0.20	5.96×10^−5^	0.03	*TCF7L2*
rs16824684	3	154914090	(T/G)	0.19	−1.00	0.25	6.04×10^−5^	0.45	*MME* (12593)
rs7234352	18	60739233	(A/G)	0.22	−0.87	0.22	8.50×10^−5^	0.23	*BCL2* (51346)
rs17819063	16	53873428	(G/A)	0.12	−1.33	0.32	4.20×10^−5^	0.70	*FTO*
rs3781093	10	8101927	(T/C)	0.16	−0.91	0.23	7.23×10^−5^	0.96	*GATA3*
rs17390295	8	2958662	(G/A)	0.05	3.13	0.79	7.46×10^−5^	0.70	*CSMD1*

*Nonsmokers*									
SNP	Chr	Position (Mb)	Allele (+/−)	MAF	Beta	SE	GENOA P value	FHS P value	Relative position (−upstream,+downstream)
rs432695	6	4674712	(A/G)	0.20	1.21	0.23	2.31×10^−7^	0.54	*intergenic*
rs4867326	5	31361704	(C/T)	0.25	1.28	0.25	4.74×10^−7^	0.61	*intergenic*
rs6873705	5	31366544	(A/G)	0.26	−1.27	0.25	4.94×10^−7^	0.60	*intergenic*
rs8080186	17	76549152	(C/G)	0.42	0.93	0.20	2.05×10^−6^	0.61	*DNAH17*
rs11649339	16	74272730	(A/C)	0.13	−1.29	0.27	2.42×10^−6^	0.99	*intergenic*
rs4462101	1	36989640	(G/A)	0.13	−1.41	0.30	2.78×10^−6^	0.99	*CSF3R* (−40761)
rs11737432	4	28767776	(G/A)	0.10	−1.38	0.30	2.93×10^−6^	0.45	*intergenic*
rs622348	13	53397564	(T/G)	0.39	−0.96	0.21	3.81×10^−6^	0.62	*intergenic*
rs17735726	18	48286557	(G/A)	0.09	−1.37	0.30	6.61×10^−6^	0.90	*MAPK4* (28361)
rs16872734	4	22484933	(G/A)	0.05	−1.91	0.42	6.97×10^−6^	0.85	*GPR125*
rs1345251	7	22248527	(G/T)	0.09	−1.43	0.32	7.25×10^−6^	0.20	*RAPGEF5*
rs6465945	7	103548958	(T/A)	0.15	1.65	0.37	9.21×10^−6^	0.73	*RELN*
rs4306080	1	25135004	(C/T)	0.27	−1.00	0.24	2.62×10^−5^	0.98	*CLIC4*
rs9521695	13	110981007	(C/G)	0.49	−1.11	0.27	4.75×10^−5^	0.10	*COL4A2*
rs2727551	7	151391653	(A/G)	0.07	1.86	0.46	5.16×10^−5^	0.09	*PRKAG2*
rs2784773	10	81919800	(T/C)	0.34	−0.91	0.23	6.84×10^−5^	0.57	*ANXA11*
rs12602288	17	68573878	(C/A)	0.49	−0.75	0.19	6.91×10^−5^	0.26	*intergenic*
rs11790127	9	18624300	(C/T)	0.10	1.38	0.35	7.95×10^−5^	0.45	*ADAMTSL1*
rs11569850	1	12164391	(G/C)	0.09	−1.36	0.35	7.83×10^−5^	0.97	*TNFRSF8*
rs6703976	1	21836934	(C/T)	0.11	1.24	0.31	5.86×10^−5^	0.88	*ALPL*
rs9893009	17	64708358	(C/G)	0.46	0.81	0.21	8.59×10^−5^	0.37	*PRKCA*

*Gene by Smoking Interactions*									
SNP	Chr	Position (Mb)	Allele (+/−)	MAF	Beta	SE	GENOA P value	FHS P value	Relative position (−upstream,+downstream)
rs16872734	4	22484933	(G/A)	0.05	3.62	0.73	3.95×10^−7^	0.24	*GPR125*
rs1395540	12	61446746	(T/C)	0.26	1.44	0.30	9.27×10^−7^	0.18	*intergenic*
rs999877	8	130490213	(G/T)	0.32	−1.36	0.29	1.63×10^−6^	0.34	*intergenic*
rs10096362	8	95083587	(G/T)	0.14	−1.83	0.40	2.21×10^−6^	0.49	*intergenic*
rs9843942	3	30729636	(G/A)	0.37	−1.24	0.27	3.00×10^−6^	0.13	*TGFBR2*
rs16951358	15	68153732	(G/C)	0.25	−1.43	0.32	4.00×10^−6^	0.02	*intergenic*
rs4776377	15	68103387	(C/T)	0.29	1.32	0.30	4.39×10^−6^	0.26	*MAP2K5*
rs7144284	14	44433152	(T/C)	0.07	2.47	0.57	7.54×10^−6^	0.48	*intergenic*
rs9574536	13	80585091	(C/T)	0.34	1.81	0.42	9.44×10^−6^	0.30	*intergenic*
rs4462101	1	36989640	(G/A)	0.13	1.78	0.43	1.83×10^−5^	0.24	*CSF3R* (−40761)
rs1679195	3	187880447	(C/T)	0.39	−1.13	0.28	3.43×10^−5^	0.08	*LPP*
rs4410439	3	64655724	(A/C)	0.63	1.02	0.27	7.05×10^−5^	0.28	*ADAMTS9*
rs1560540	13	75882495	(G/A)	0.52	−1.05	0.28	6.86×10^−5^	0.14	*TBC1D4*
rs2618157	15	39844315	(G/A)	0.09	−2.22	0.57	5.31×10^−5^	0.18	*THBS1* (−28812)
rs7008465	8	1722760	(T/G)	0.15	−1.52	0.38	3.36×10^−5^	0.40	*CLN8*
rs6565497	17	78912633	(A/T)	0.57	−1.13	0.27	1.17×10^−5^	0.29	*RPTOR*
rs4867326	5	31361704	(T/C)	0.75	1.32	0.32	2.31×10^−5^	0.46	*CDH6* (32451)
rs6032769	20	44938851	(C/T)	0.22	1.52	0.36	1.26×10^−5^	0.16	*CDH22* (−1714)
rs10514838	9	123746796	(T/C)	0.10	−1.83	0.48	6.40×10^−5^	0.14	*C5*

In addition, GENOA SNPs located in genes that are related to CAC with p-values between 10^−4^ and 10^−5^ are shown (used in secondary analysis). The corresponding Framingham p values for GENOA index SNP are presented.

SNP, single nucleotide polymorphism; Chr, chromosome; Mb, megabases; MAF, minor allele frequency; Beta, beta coefficient; SE, standard error. Models were adjusted for the following covariates: age, sex, body mass index (BMI), pulse pressure, diabetes, systolic blood pressure (SBP), use of anti-hypertensive medications, use of lipid lowering medications, and LDL-cholesterol.

We conducted secondary analysis to investigate gene regions containing SNPs that showed suggestive associations in GENOA (p≤10^−5^) by testing multiple SNPs in FHS sibships within +/−250 kb of GENOA index SNPs [21]. We also included genes containing SNPs with p-values between 10^−4^ and 10^−5^ in GENOA with known functions relevant to CAC (Table 2). Table 3 shows the results for FHS subgroups for SNPs that reached significance thresholds using an LD-based Bonferroni approach to correct for multiple testing (p≤0.05/number of LD blocks in each gene region), thus avoiding potential over-correction for correlated SNPs [19].

**Table 3:** Secondary analysis: results of gene-based replication in FHS for genes containing SNPs associated with CAC in the GENOA discovery cohort.

*Smokers*										
Locus				Association signal					Nearby genes	
SNP	Chr	Position (Mb)	Allele (+/−)	MAF	Beta	SE	P value	# of blocks (threshold)	Relative position (−upstream,+downstream)	Cohort
rs8047995	16	78949736	(G/C)	0.32	1.13	0.25	4.51×10^−6^		*WWOX*	GENOA
rs10492908	16	79006863	(A/G)	0.05	1.4	0.45	1.70×10^−3^	50 (1.0×10^−3^)	*WWOX*	FHS
rs10128255	10	114742835	(G/A)	0.36	0.82	0.20	6.0×10^−5^		*TCF7L2*	GENOA
rs11196175	10	114736614	(C/T)	0.33	0.72	0.21	4.0×10^−4^	30 (1.7×10^−3^)	*TCF7L2*	FHS

*Nonsmokers*										
SNP	Chr	Position (Mb)	Allele (+/−)	MAF	Beta	SE	P value	# of blocks (threshold)	Relative position (−upstream,+downstream)	Cohort
rs2727551	7	151391653	(A/G)	0.07	1.86	0.46	5.2×10^−5^		*PRKAG2*	GENOA
rs2727527	7	151347462	(A/C)	0.3	−0.67	0.20	9.0×10^−4^	27 (1.9×10^−3^)	*PRKAG2*	FHS
rs11569850	1	12164391	(G/C)	0.09	−1.36	0.35	7.8×10^−5^		*TNFRSF8*	GENOA
rs4304595	1	11946389	(A/C)	0.31	−0.78	0.21	2.0×10^−4^	21 (2.4×10^−3^)	*PLOD1* (upstream)	FHS

*Gene by Smoking Interaction*										
SNP	Chr	Position (Mb)	Allele (+/−)	MAF	Beta	SE	P value	# of blocks (threshold)	Relative position (−upstream,+downstream)	Cohort
rs4462101	1	36989640	(G/A)	0.13	1.78	0.43	1.8×10^−5^		*CSF3R* (−40761)	GENOA
rs7528341	1	37141174	(C/T)	0.21	0.95	0.29	4.0×10^−4^	30 (1.7×10^−3^)	*FTLP18* (44716)	FHS
rs1679195	3	187880447	(C/T)	0.39	−1.13	0.28	3.4×10^−5^		*LPP*	GENOA
rs9871228	3	187795325	(G/T)	0.23	−1.36	0.42	6.2×10^−4^	38 (1.3×10^−3^)	*LPP* (−75747)	FHS
rs4410439	3	64655724	(A/C)	0.37	1.02	0.27	7.1×10^−5^		*ADAMTS9*	GENOA
rs4688504	3	64684941	(C/T)	0.47	0.78	0.21	1.0×10^−4^	30 (1.7×10^−3^)	*ADAMTS9*	FHS
rs1560540	13	75882495	(G/A)	0.48	1.05	0.28	6.9×10^−5^		*TBC1D4*	GENOA
rs1062087	13	75884216	(C/T)	0.12	1.91	0.38	4.2×10^−7^	40 (1.3×10^−3^)	*TBC1D4*	FHS
rs7008465	8	1722760	(T/G)	0.15	−1.52	0.38	3.4×10^−5^		*CLN8*	GENOA
rs3758014	8	1849275	(A/G)	0.28	−1.97	0.52	7.8×10^−5^	45 (1.1×10^−3^)	*ARHGEF10*	FHS

The number of LD blocks in each gene region used to correct for multiple testing are presented, as well as thresholds for significance in FHS.

SNP, single nucleotide polymorphism; Chr, chromosome; Mb, megabases; MAF, minor allele frequency; Beta, beta coefficient; SE, standard error. Models were adjusted for the following covariates: age, sex, body mass index (BMI), pulse pressure, diabetes, systolic blood pressure (SBP), use of anti-hypertensive medications, use of lipid lowering medications, and LDL-cholesterol.

In GENOA, rs8047995 in intron 8 of the gene for WW domain-containing oxidoreductase (*WWOX*) showed associations only in smokers (p = 4.5×10^−6^). A different variant in intron 8 of *WWOX* (rs10492908) also showed associations only in smokers in FHS (p = 1.7×10^−3^). Also in GENOA, rs10128255 in the gene for transcription factor 7 like-2 (*TCF7L2*) showed associations only in smokers (Tables 2 and 3, p = 6.0×10^−5^), and another *TCF7L2* SNP (rs11196175) showed associations in FHS smokers (Table 3 p = 4.0×10^−4^). In GENOA nonsmokers, we found associations with rs11569850 (p = 7.8×10^−5^) in the gene for tumor necrosis factor receptor superfamily, member 8 (*TNFRSF8*).

**Figure 1:**
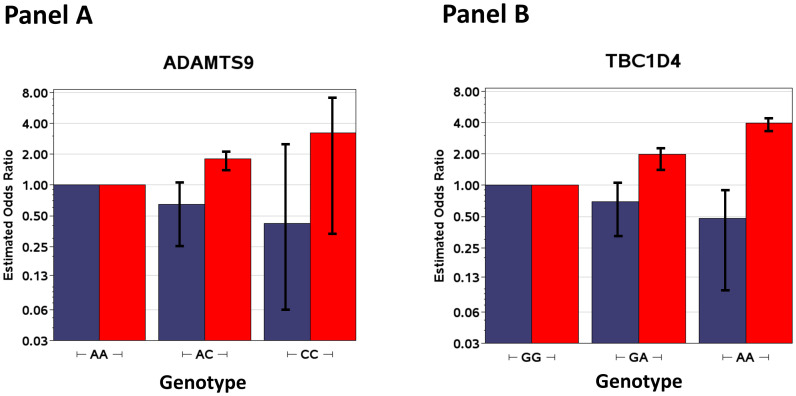
GxS interaction effects stratified by smoking status for *ADAMST9* (rs4410439) (Panel A) and *TBC1D4* (rs1560540) (Panel B) genotypes. The Figure shows the additive genotype effects (odds ratios) for each smoking strata used to calculate interaction tests (blue bars for nonsmokers and red bars for smokers). The odds ratios on the y-axis are plotted on the log scale with error bars for 95% confidence intervals, and the genotypes are shown on the x-axis.

For GxS interactions, rs4410439 in the gene for ADAM metallopeptidase thrombospondin type 1 motif, 9 (*ADAMTS9*) showed associations in GENOA (Table 3, p = 7.1×10^−5^). We also found associations with *ADAMTS9* (rs4688504) in the FHS cohort (p = 1.0×10^−4^). Another variant (rs1560540) that showed associations for GxS interaction in GENOA (p = 6.9×10^−5^) was located in the gene for TBC1 domain family member 4 (*TBC1D4*). We found stronger associations with *TBC1D4* (rs1062087) in the FHS cohort (p = 4.2×10^−7^). Figure 1 shows the stratified effects for genotypes for *ADAMST9* (rs4410439) and *TBC1D4* (rs1560540) from the GxS interaction results in Table 3. Panel A shows the additive genotype effects (odds ratios obtained by exponentiation of the beta coefficients) for each smoking strata used to calculate the interaction test for rs4410439 in *ADAMST9*. For smokers (β = 0.59, SE = 0.17, p = 4.5×10^−4^), the odds ratio (OR) for reference AA genotypes was set at 1, the OR for AC genotypes was 1.80 (95% confidence interval of 1.47–2.13), and the OR for CC genotypes was 3.23 (0.35–7.40). For nonsmokers (β = −0.43, SE = 0.21, p = 3.8×10^−2^), the OR for AC genotypes was 0.65 (0.24–1.06) and the OR for CC genotypes was 0.42 (0.06–2.49). Likewise, Panel B shows the additive genotype effects by smoking strata for rs1560540 in *TBC1D4*. For smokers (β = 0.69, SE = 0.20, p = 4.7×10^−4^), the OR for reference GG genotypes was set at 1, the OR for GA genotypes was 1.99 (1.60–2.38), and the OR for AA genotypes was 3.96 (3.58–4.35). For nonsmokers (β = −0.36, SE = 0.19, p = 5.8×10^−2^), the OR for GA genotypes was 0.69 (0.32–1.07) and the OR for AA genotypes was 0.48 (0.10–0.86). For both genes, the direction of effects are opposite for the smokers versus nonsmokers (βs of opposite signs), the hallmark of GxE interaction.

We compared the results of the GxS interaction GWAS with the recent GWAS meta-analysis for main SNP effects on CAC quantity [10]. We interrogated the SNPs with the strongest associations in the GWAS meta-analysis including rs1333049 (*CDKN2B*), rs9349379 and rs2026458 (*PHACTR1*), rs3809346 (*COL4A2*), rs6783981 (*SERPIN1*), rs17676451 (*HAL*), rs6604023 (*CDC7*), and rs8001186 (*IRS2*). However, we did not find significant associations with any of these loci for GxS interaction (GENOA discovery cohort) with nominal p-values ranging from 0.09 to 0.49.

## Discussion

We used a genome-wide approach to investigate GxS interactions to identify genetic variants associated with CAC exclusively in either smokers or nonsmokers, and with GxS interactions in the GENOA cohort. Our primary analysis used GWAS in GENOA, followed by attempts to replicate associated index SNPs (p≤10^−5^) in FHS. However, these tests did not yield SNP associations that met genome-wide significance in GENOA, or standard SNP-based replication in FHS (Table 2). Primary analyses were likely limited by the relatively small sample size of the discovery GENOA cohort that reduced statistical power for main effects in GWAS results within strata. In secondary analysis, we used a gene-based approach, testing multiple SNPs in FHS within associated gene regions (+/−250 kb of GENOA index SNPs) [19]. We found many instances of SNPs within gene regions that showed significant associations in FHS after correction for multiple testing using numbers of LD blocks in each region (Table 3). Overall, these genes were not represented by identical SNPs in the two cohorts, likely due to differences in allele frequencies or functional differences of SNPs in different regions of the same gene. In some instances, we observed different direction of effects (beta-coefficients) for different SNPs in the same gene (Table 3). Using theoretical modeling, Lin and coworkers demonstrated valid “flip-flop” associations may occur even for identical SNPs due to correlations with other causal variants that differ among cohorts because of interaction effects or differences in LD patterns caused by sampling variation within ethnic groups or evolutionary history between ethnic groups [22]. In general, replication in GxE GWAS may prove challenging, given the complex nature of traits like CAC that are influenced by numerous interactions among multiple loci and environmental factors, as well as differences among cohorts in LD structure and environmental exposures.

Our GWAS studies of GxS interactions identified several genes that showed concordance of results in GENOA and FHS that are involved in diverse cellular processes such as inflammation and osteogenesis that are relevant to CAC. In particular, Inflammation is a likely mediator of GxS interactions, since many of the deleterious effects of smoking are due to induction of inflammatory responses, contributing to chronic diseases such as CHD [23]. Inflammatory markers are also well known risk factors for type 2 diabetes (T2D), providing a likely physiological connection between development of CHD and T2D [24]. Recent *in vitro* experiments in human umbilical vein endothelial cells demonstrated that nicotine stimulates cellular inflammatory response via activation of the NF-κB transcription factor axis by a second messenger pathway [25]. In a rat model, exposure to cigarette smoke caused changes in levels of inflammatory markers including NF-κB in cardiac tissues [26]. In addition to inflammation, the NF-κB axis plays a central role in CAC quantity and bone remodeling by induction of osteoclast differentiation [27], [28].

In GENOA and FHS, the gene for transcription factor 7 like-2 (*TCF7L2*) showed associations only in smokers (Table 3). *TCF7L2* (chromosome 10) has shown associations with T2D in a previous GWAS [29], and may impair pancreatic beta-cell function with effects on blood glucose homeostasis [30]. *TCF7L2* has shown associations with angiographically determined CHD in diabetic and non-diabetic patients [31], as well as with CVD, ischemic stroke, peripheral artery disease, and all-cause mortality [32]. *TCF7L2* encodes the Tcf-4 transcription factor in the Wnt Signalling pathway, directly regulating beta-catenin, a major activator of the NF-κB axis [33]. Activation of the NF-κB axis may provide a clue concerning the role of *TCF7L2* in CAC development, since osteogenesis and bone remodeling are regulated by NF-κB [27], [28].

The gene for WW domain-containing oxidoreductase (*WWOX*) also showed associations only in smokers from both GENOA and FHS (Table 3). *WWOX* (chromosome16) is an established tumor suppressor gene that is associated with CHD, bone development, and higher methylation levels in smokers [34]. In previous population-based studies, variants in *WWOX* have shown associations with CHD and left ventricular mass [35], [36]. Ablation of *WWOX* in knock-out mouse strains (*Wwox* -/-) caused development of osteosarcomas, as well as osteopenia and bone growth retardation [34], [37].

In GENOA nonsmokers, we found associations in the gene for tumor necrosis factor receptor superfamily, member 8 (*TNFRSF8*) (Table 3). Like *TCF7L2*, this gene may also influence CAC via the NF-κB axis that regulates inflammatory response and bone remodeling [27], [28]. *TNFRSF8* is a member of the TNF-receptor superfamily that play key roles in signaling pathways that regulate NF-κB activation via interaction with TNF cytokines. In FHS, the associated variant in this chromosomal region was in an intergenic region near *PLOD1* (rs4304595, p = 2.0×10^−4^) (Table 3).

For GxS interactions, rs1062087 within the gene for TBC1 domain family member 4 (*TBC1D4*) showed stronger association in FHS than the SNP identified in GENOA (Table 3). Interestingly, rs1062087 is a nonsynonymous variant (Ile818Val) that is located in the same LD block with rs1560540 that showed associations in GENOA. *TBC1D4* encodes the AS160 protein that mediates insulin homeostasis by regulating glucose uptake in fat and muscle cells via GLUT4 glucose transporters. Inflammatory markers (TNF-α, IL-1, IL-6) are associated with a reduction of AS160 activities, resulting in increased insulin resistance [38]. Another variant (rs4410439) that showed associations for GxS interactions in GENOA (P = 7.1×10^−5^) was located in the gene for ADAM metallopeptidase thrombospondin type 1 motif, 9 (*ADAMTS9*) (Table 3). We also found associations with *ADAMTS9* (rs4688504) in the FHS cohort (p = 1.0×10^−4^) (Table 3). *ADAMTS9* encodes a metalloprotease that is involved in thrombosis, cleaving veriscan, proteoglycans, and aggrecan. In transgenic mice that were heterozygous for an inactivated allele carrying the *LacZ* reporter gene (*Adamts9^+LacZ^*), *ADAMTS9* haploinsufficiency altered cardiovascular development and allostasis, resulting in valvular and aortic anomalies [39]. As with *TCF7L2*, both *TBC1D4* and *ADAMTS9* have shown associations in GWAS meta-analysis for T2D [29], [40]. Such genes may provide new insights into the well-known relationship between CHD and T2D, perhaps mediated by inflammatory processes.

Perhaps the future best use of existing GWAS data from epidemiological cohorts is the identification of loci involved in interactions (gene by gene, gene by environment) that underlie complex diseases such as CHD and their risk factors. Our results demonstrate that such interactions (i.e., gene by smoking) may be generalizable among cohorts, given that many of the genes identified in GENOA also showed significant associations in FHS. These interactions are likely to reflect the role of particular metabolic or physiological pathways that include many genes, and that interact with environmental factors such as smoking. In our study, we found that many of the replicated genes were involved in inflammatory pathways mediated by the NF-κB axis. In addition, three of the loci associated with CAC also showed associations in GWAS meta-analysis of T2D [40], a chronic disease with altered inflammatory pathways and increased CHD risk. Since smoking may cause chronic and systemic inflammation, association of these genes in GENOA likely reflect their roles in CAC development and progression via participation in inflammatory pathways. Interestingly, the NF-κB axis also regulates bone remodeling, providing a link between inflammation and pathways of osteogenesis involved in development and progression of CAC. Additional genetic studies will be required for further tests of these genes in other human populations, as well as functional studies to understand how these genes influence gene by smoking interactions.
