# Correction: Genome-Wide Association Study of Gene by Smoking Interactions in Coronary Artery Calcification

**DOI:** 10.1371/journal.pone.0093722

**Published:** 2014-04-03

**Authors:** The PLOS ONE Staff

In the Methods section, under Data Analysis, there is an error in the equation. The equation listed in both the online and PDF versions of this article is incorrect. Please view the complete, correct equation here:

(β_smokers_– β_nonsmokers_) / √ (se_smokers_^2^+se_nonsmokers_^2^)
